# (No) time for control: Frontal theta dynamics reveal the cost of temporally guided conflict anticipation

**DOI:** 10.3758/s13415-015-0367-2

**Published:** 2015-06-26

**Authors:** Joram van Driel, Jennifer C. Swart, Tobias Egner, K. Richard Ridderinkhof, Michael X Cohen

**Affiliations:** Department of Psychology, University of Amsterdam, 1018 XA Amsterdam, The Netherlands; Amsterdam Brain and Cognition, University of Amsterdam, Amsterdam, The Netherlands; Department of Cognitive Psychology, Vrije Universiteit Amsterdam, Amsterdam, The Netherlands; Donders Centre for Cognitive Neuroimaging, Radboud University Nijmegen, Nijmegen, The Netherlands; Department of Psychology and Neuroscience and Center for Cognitive Neuroscience, Duke University, Durham, NC USA

**Keywords:** Cognitive control, Functional connectivity, Timing, Oscillatory dynamics

## Abstract

During situations of response conflict, cognitive control is characterized by prefrontal theta-band (3- to 8-Hz) activity. It has been shown that cognitive control can be triggered proactively by contextual cues that predict conflict. Here, we investigated whether a pretrial preparation interval could serve as such a cue. This would show that the temporal contingencies embedded in the task can be used to anticipate upcoming conflict. To this end, we recorded electroencephalography (EEG) from 30 human subjects while they performed a version of a Simon task in which the duration of a fixation cross between trials predicted whether the next trial would contain response conflict. Both their behavior and EEG activity showed a consistent but unexpected pattern of results: The conflict effect (increased reaction times and decreased accuracy on conflict as compared to nonconflict trials) was stronger when conflict was cued, and this was associated with stronger conflict-related midfrontal theta activity and functional connectivity. Interestingly, intervals that predicted conflict did show a pretarget increase in midfrontal theta power. These findings suggest that temporally guided expectations of conflict do heighten conflict anticipation, but also lead to less efficiently applied reactive control. We further explored this post-hoc interpretation by means of three behavioral follow-up experiments, in which we used nontemporal cues, semantically informative cues, and neutral cues. Together, this body of results suggests that the counterintuitive cost of conflict cueing may not be uniquely related to the temporal domain, but may instead be related to the implicitness and validity of the cue.

*Cognitive control* refers to a set of mental capacities devoted to optimize goal-directed behavior in situations of multiple competing response alternatives (Botvinick, Braver, Barch, Carter, & Cohen, 2001; Ridderinkhof, Forstmann, Wylie, Burle, & van den Wildenberg, 2010; Ridderinkhof, Ullsperger, Crone, & Nieuwenhuis, 2004). Neuroscience has tied these adaptive control functions to processes in frontal brain networks (Fuster, 2000; E. K. Miller, 2000), where the medial frontal cortex (MFC) is thought to signal the need for control in response to challenging situations (Alexander & Brown, 2011; Botvinick, Cohen, & Carter, 2004; Ito, Stuphorn, Brown, & Schall, 2003), which is communicated to the dorsolateral prefrontal cortex (DLPFC; MacDonald, Cohen, Stenger, & Carter, 2000). Both of these regions exert top-down influence over lower, task-related sensorimotor processing (Cohen, van Gaal, Ridderinkhof, & Lamme, 2009; Danielmeier, Eichele, Forstmann, Tittgemeyer, & Ullsperger, 2011; Egner & Hirsch, 2005; B. T. Miller & D’Esposito, 2005), in order to adjust future behavior (Kerns et al., 2004). Cognitive electrophysiology has provided compelling evidence of theta-band (3- to 8-Hz) oscillatory activity as the underlying “language” of communication within this network (see Cavanagh & Frank, 2014, and Cohen, 2014, for reviews), where the MFC has been proposed to be a “hub” for theta phase-synchronized information transfer (Cohen, 2011).

Cognitive control is a transient response, waxing and waning depending on the presence or absence of risks or demands such as response conflict. Indeed, because frontally mediated cognitive control is effortful, it is inefficient to recruit these mechanisms continuously (Ridderinkhof, Ullsperger, et al., 2004). Here, *conflict* is defined as the incongruence between a task-relevant learned response and a task-irrelevant stimulus feature, which results in slower and more error-prone behavior relative to nonconflict (the “conflict effect”). The immediate trial history in a typical conflict task influences the level of activated control in the subsequent trial (the “congruency sequence effect” [CSE]; Egner, 2007; Gratton, Coles, & Donchin, 1992). When the previous trial imposed conflict, the cognitive control system is engaged, resulting in better performance on the subsequent conflict trial. Importantly, these trial-to-trial fluctuations in behavioral conflict effects have been shown to covary with trial-to-trial variability in midfrontal theta activity (Cohen & Cavanagh, 2011).

Although the CSE could be regarded as a form of anticipatory control through conflict adaptation (Botvinick et al., 2001; Egner, 2007), it is still *reactive* in nature (Ridderinkhof et al., 2010): Earlier conflict detection boosts adaptive control, as called forth by conflict encountered on the subsequent trial. Anticipatory control can be triggered *proactively* as well, by means of contextual cues (Gratton et al., 1992). For example, when an informative red cross-sign symbol was presented always before an incongruent arrow-flanker target, performance improved relative to when an uninformative question-mark symbol was used as the cue (Correa, Rao, & Nobre, 2009). Interestingly, this behavioral cueing effect was accompanied by the attenuation of a frontal N2 component (see also Strack, Kaufmann, Kehrer, Brandt, & Stürmer, 2013), a potential neural marker of conflict processing (van Veen & Carter, 2002; cf. Cohen & Donner, 2013). Similar cueing effects have been found with other conflict tasks and type of cues, ranging from semantically informative word cues preceding the target (Alpay, Goerke, & Stürmer, 2009; Wühr & Kunde, 2008), to the spatial location (Corballis & Gratton, 2003; Crump, Gong, & Milliken, 2006) or color (Lehle & Hübner, 2008) of the target itself.

One potentially relevant source of contextual information has received surprisingly little attention in the conflict-cueing literature: time. Temporal contingencies between events are ubiquitous in our natural environment and provide information about which actions to take and when. For example, while approaching a traffic light, seeing it change from green to yellow triggers a cascade of temporal predictions (e.g., *how long* is the light going to be yellow before it turns red, *when* will I arrive at the traffic light given my speed?), which ultimately results in a decision: Should I stop or not? The literature on temporal orienting has shown that temporally predictable stimuli trigger time-dependent preparatory neural dynamics, as well as faster and more accurate behavioral responses (see Nobre, Correa, & Coull, 2007, for a review). For example, in a color–word Stroop task in which the intervals between the irrelevant (color) and relevant (word) stimulus dimensions were predictable rather than random, subjects were able to strategically allocate attention in time to reduce the cost of Stroop interference (Appelbaum, Boehler, Won, Davis, & Woldorff, 2012). Although these accounts relate to temporal predictions about when a stimulus will occur and when to respond to it, it is less clear whether temporal predictions can be made about when *conflict* is most likely to occur. In other words, can temporal information be used as a cue to predict conflict?

To our knowledge, only one prior study has specifically addressed this question. In a letter-flanker task, Wendt and Kiesel (2011) varied the contingencies between the proportion of incongruent trials and the duration of a pretarget fixation cross (the “warning signal,” also sometimes called the “foreperiod”), such that subjects could predict the likelihood of upcoming conflict on the basis of temporal information. According to the authors, this is a purely *endogenous* form of proactive control: The internally generated estimation of the fixation-cross duration provides the conflict-predicting information, not the exogenous presentation of the fixation-cross per se. Their behavioral findings seemed to indicate that subjects were indeed able to use these temporal cues to prepare for upcoming conflict, but only when long (1,200-ms) instead of short (200-ms) durations were associated with a high probability of conflict.

The goal of the present study was twofold. First, we aimed to replicate the temporal-cueing effect observed by Wendt and Kiesel (2011), using another type of conflict task. Second, we reasoned that measuring electroencephalography (EEG) while using temporal cues would provide a valuable tool to investigate the online neural dynamics of cognitive control during temporal conflict anticipation. Here, we used a color–location Simon task (Simon & Rudell, 1967), in which we manipulated the duration of a pretarget fixation cross so as to predict with 80 % validity the congruency of the upcoming trial, analogous to the Wendt and Kiesel paradigm. Specifically, we hypothesized that (1) the behavioral conflict effect would reduce when conflict rather than nonconflict was cued, and (2) that conflict-related midfrontal theta activity, often observed as being locked to the response (Cohen & Cavanagh, 2011), would shift to the pretarget conflict-predicting intervals. Finally, we assessed in three follow-up behavioral experiments whether our findings extended to (i) nontemporal symbolic cues, (ii) explicit word cues, and (iii) noninformative versus deterministic cues.

## Materials and method

### Subjects

For the EEG experiment, 34 subjects participated in exchange for €20 or course credit. The data of four subjects were excluded because of excessive muscle artifacts in the EEG signal, problems during the EEG recording, or performance at chance level (accuracy ~50 %) in one or more blocks of the task. Thus, in total the data of 30 subjects were included in the analyses (age range 19–32 years, *M* = 22.9; 24 females, six males, two left-handed). For the three follow-up behavioral experiments, a total of 51 subjects participated (Follow-Up Exp. 1: *N* = 20, 14 females, six males; age range 18–31 years, *M* = 22.2; Exp. 2: *N* = 16, ten females, six males; age range 19–24 years, *M* = 21.4; Exp. 3: *N* = 15, 12 females, three males; age range 18–30 years, *M* = 23.3). One subject was excluded from the analysis of Follow-Up Experiment 2 because of chance-level performance. In all experiments, the subjects did not report neurological and psychiatric disorders or the use of psychotropic drugs, and all reported having normal or corrected-to-normal vision. Subjects signed an informed consent form before participation. The experiments were approved by the local ethics committee, and all procedures complied with the relevant laws and institutional guidelines.

### Task

In all experiments, subjects performed a modified version of the Simon task. In each trial, a colored circle (hereafter referred to as the “target”) appeared on a light grey screen. Subjects were instructed to respond as quickly as possible with their left thumb for blue and yellow targets, and with their right thumb for red and green targets (or vice versa; color–response mapping was counterbalanced across subjects). Targets subtended 0.70 deg of visual angle (dva) and appeared for 100 ms at 5.02 dva left or right from a fixation point, which consisted of a small dark gray square of 0.10 dva in the center of the screen. Trials ended upon responding or after a response window of 1,000 ms had passed, in which case feedback on response speed was presented, with the words “respond faster!” The trial end was followed by an intertrial interval of 1,000 ms, during which the fixation point remained on screen.

Response conflict was induced on incongruent trials, in which the location of the target corresponded to the spatially incompatible response hand (e.g., if the blue target, which required a left-hand response, appeared right of the fixation point). On congruent trials, the target location always corresponded to the spatially compatible response hand. One half of trials were incongruent, and congruent and incongruent trials were presented in random order (see below for randomization procedure).

Each trial started with a nonconflict pretarget cue that predicted the congruency of the upcoming trial. In one EEG experiment, and three follow-up behavioral experiments, we manipulated the nature of this pretarget cue. First we describe the EEG experiment in depth, followed by our EEG measurement and analysis approach. At the end of this section, we then describe the follow-up experiments.

In the EEG experiment, the pretarget cue was a white fixation cross (0.55 dva), superimposed upon the fixation point, of variable duration. From here on we refer to this fixation cross as the “warning signal” (WS). The WS duration could be either short (400 ms) or long (1,400 ms), after which the fixation point reappeared for 300 ms (the “pretarget interval” or PTI; see below), followed by the target.

**Fig. 1:**
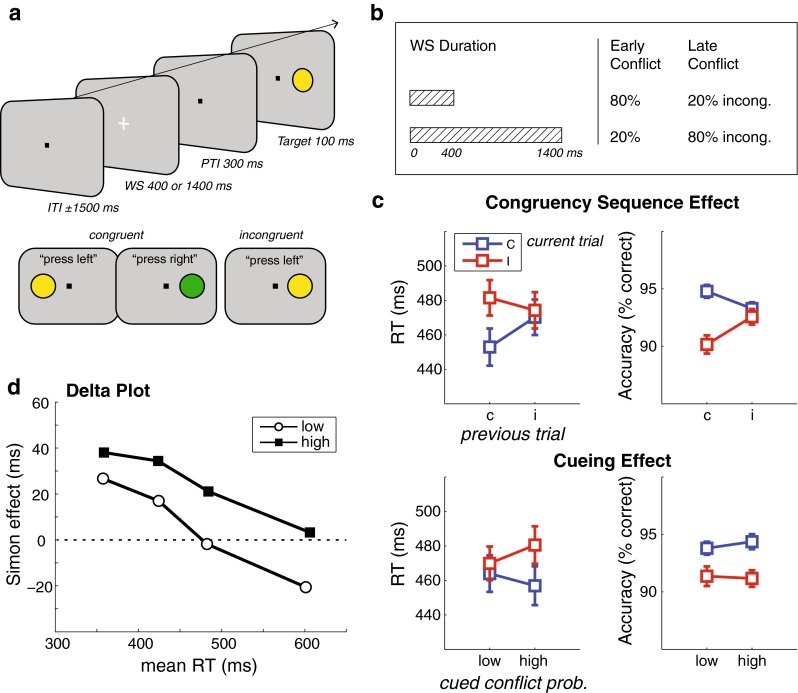
Task paradigm and behavioral results. (**a**) Trial types, stimulus sequence, and timing (top), and examples of congruent and incongruent trials (bottom). (**b**) The proportions of congruent/incongruent trials depended on the duration of the warning signal (WS). (**c**) Reaction times (RTs, left) and accuracy (right) for congruent (C) and incongruent (I) trials as a function of previous trial congruency (top) and cued conflict probability (bottom). Error bars represent ±1 *SEM*. (**d**) Delta plot showing conflict effects (current trial RT incongruent – congruent) as a function of quartiles of the RT distribution, for both low (white open circles) and high (black solid squares) conflict probability, collapsed over groups (i.e., irrespective of WS duration)

Crucially, the association between WS duration and trial congruency was determined by an experimental between-subjects condition: 15 subjects performed the early-conflict condition, in which 80 % of the short WSs were followed by incongruent trials (indicating high conflict probability), and 20 % of the long WSs were followed by incongruent trials (indicating low conflict probability). The other 15 subjects performed the late-conflict condition, in which these proportions were reversed: 20 % of the short WSs were followed by incongruent trials (low conflict probability), and 80 % of the long WSs were followed by incongruent trials (high conflict probability). In both conditions, the short and long WSs were presented equally often, keeping the overall proportion of incongruent trials at 50 %, and the temporal expectation of target occurrence balanced. The order of congruent and incongruent trials, together with the order of the WSs, was pseudorandomized, such that there was never a repetition of the same combination of WS, trial type, and stimulus properties (e.g., two consecutive times an incongruent trial with a blue circle presented on the left, preceded by a 1,400-ms WS). See Fig. 1a and b for an overview of the experimental design.

The primary motivation for using a between-subjects design was to avoid transfer effects of, or switch costs between, the learned association between WS and conflict across conditions. That is, the association is opposite in the early- and late-conflict conditions, and subjects were uninformed about the nature of the association (see below). Moreover, the between-subjects manipulation allowed us to increase the number of trials per condition. Although we acknowledge the potential limitations of a between-subjects design (i.e., low numbers of subjects per early-/late-conflict condition, and possible group differences that were unaccounted for), we believe that these do not outweigh the importance of controlling for transfer effects and switch costs. Moreover, and to foreshadow some of our results, the between-subjects factor Group did not show interaction effects with either conflict or conflict probability.

The motivation for using an additional PTI of a fixed 300 ms after the WS was to temporally isolate the WS, thereby making it more salient. Moreover, we reasoned that the PTI would control for confounding effects of temporal orienting (Nobre et al., 2007). That is, both after a short WS of 400 ms and a long WS of 1,400 ms, the target always appeared 300 ms after PTI onset. Thus, even though conditions differed in total pretarget duration, the PTI was meant to “reset” a temporal hazard function of target onset (inferring the probability of target onset given that it has not yet occurred) across all conditions. In contrast, uncertainty remained with respect to conflict: For instance, after 400 ms during the long WS in the late-conflict condition, the upcoming trial was only an incongruent conflict trial in 80 % of the time. In other words, we were interested in conflict expectation, not stimulus occurrence expectation.

Before the start of the experiment, subjects were informed about the different durations of the fixation cross, but not about the association of these durations with congruency. Subjects completed one practice block of 50 trials during which feedback on accuracy (“Correct,” “Incorrect”) was provided upon response in each trial. In the practice block, the temporal cues had a validity of 100 %, to enhance learning of the cue–conflict contingencies. The main task consisted of ten blocks of 100 trials. Between consecutive blocks, there were self-paced breaks during which feedback on task performance (average reaction time and accuracy) was shown on screen. After the main task, subjects were asked whether they noticed the duration–conflict associations, and if so, whether they had used a particular strategy in preparing for conflict based on the temporal cues. Although this was not assessed quantitatively, one subject indeed noticed the association, but did not report having used a particular strategy. One other subject noticed that “there was something about” the WS durations, but could not formulate what. Yet another subject who performed in the early-conflict condition did not notice the WS–conflict association in particular, but did report having used the strategy of trying to pay more attention and respond faster when the target came early. All other subjects explicitly reported not having noticed the cue manipulation, nor having used any strategy.

### EEG data collection and preprocessing

During this first experiment, EEG data were acquired at 512 Hz from 64 channels (using a BioSemi ActiveTwo system; http://biosemi.com) placed according to the international 10–20 system, under and above the left eye for vertical EOG, to the left and right sides of the left and right eyes, respectively, for the horizontal electrooculogram (EOG), and from both earlobes for referencing. Offline, the EEG data were high-pass filtered at 0.5 Hz and epoched from –3.2 to 2 s, locked to target onset. These wide ranges avoided edge artifacts resulting from time–frequency decomposition (see below). All epochs were linearly baseline-corrected with a 200-ms pretarget baseline and visually inspected for artifacts. Those epochs containing electromyographic or other artifacts not related to eye blinks were manually removed, resulting in an average of 63 rejected epochs per participant (*SD* = 40). On the resulting epochs, an independent component analysis was performed with the EEGLAB software package (Delorme & Makeig, 2004) in MATLAB (The MathWorks). Components related to eye blinks or artifacts in the signal that could be clearly distinguished from brain activity were removed from the data. On average 1.33 components (range = 1–4) were removed. The EOG signal was included in the independent component analysis but was left out of the further analyses. Next, the surface Laplacian of the EEG data was estimated (Perrin, Pernier, Bertrand, & Echallier, 1989), which is equivalent to the current source density approach (Kayser & Tenke, 2006). This method has previously been applied for sharpening EEG topography and performing synchronization analyses (Cavanagh et al., 2010; Cohen et al., 2009; van Driel, Ridderinkhof, & Cohen, 2012). The Laplacian accentuates local effects while filtering out distant effects due to volume conduction (i.e., deeper brain sources that project onto multiple electrodes, thereby obscuring neurocognitively modulated long-range functional connectivity; Oostendorp & Oosterom, 1996; Srinivasan, Winter, Ding, & Nunez, 2007; Winter, Nunez, Ding, & Srinivasan, 2007). For estimating the surface Laplacian, we used a 10th-order Legendre polynomial, and lambda was set at 10^–5^.

### EEG time–frequency decomposition

The target-locked epoched EEG time series were decomposed into their time–frequency representations with custom-written MATLAB scripts, by convolving them with a set of Morlet wavelets with frequencies ranging from 1 to 50 Hz in 40 logarithmically scaled steps. These complex wavelets were created by multiplying perfect sine waves (sine wave = *e*^*i*2π*ft*^, where *i* is the complex operator, *f* is the frequency, and *t* is time) with a Gaussian (Gaussian = *e*^–*t*2/2*s*2^, where *s* is the width of the Gaussian). The width of the Gaussian was set to four cycles [*s* = 4/(2π*f*)], in order to have a good trade-off between temporal and frequency resolution. The fast Fourier transform (FFT) was applied to both the EEG data and the Morlet wavelets, and these were then multiplied in the frequency domain (equivalent to convolution in the time domain), after which the inverse FFT was applied. From the resulting complex signal *Z*_*t*_ (down-sampled to 40 Hz), an estimate of frequency-specific power at each time point was defined as [real(*Z*_*t*_)^2^ + imag(*Z*_*t*_)^2^], and an estimate of the frequency-specific phase at each time point was defined as arctan[imag(*Z*_*t*_) / real(*Z*_*t*_)]. The trial-averaged power was decibel normalized (dB Power_*tf*_ = 10 * Log_10_[Power_*tf*_ / Baseline Power_*f*_]), where for each channel and frequency the condition-averaged power signal during an interval of –250 to –50 ms relative to WS onset served as the baseline activity.

Intersite phase clustering (ISPC) measures the similarity between pairs of channels of their time–frequency phase values across trials. This measure of phase synchronization is thought to reflect interregional functional connectivity (Fries, 2005; Siegel, Donner, & Engel, 2012). ISPC is computed as follows:

$$ ISP{C}_{tf}=\left|\frac{1}{N}\times {\displaystyle \sum_{n=1}^N{e}^i\left({\phi}_{j,tf}-{\phi}_{k,tf}\right)}\right|, $$

where *N* is the number of trials, *n* is the trial number, *ϕ* is the phase angle, and *k* and *j* are the two channels. ISPC can range from 0 (*no phase synchrony between channels*) to 1 (*identical phase angles between channels*) for each time–frequency (*tf*) point. ISPC values were baseline transformed into percent signal change (100 * [(ISPC_*tf*_ – ISPC*base*_*f*_) / ISPC*base*_*f*_]), using the same baseline time window as for trial-averaged power. We used a condition-specific baseline for ISPC in order to control for spurious results induced by differences in the number of trials for the different trial types. Time–frequency decomposition was performed both target-locked and response-locked (i.e., the time series of each EEG epoch were re-sorted to be time-locked to the buttonpress).

### Electrodes, frequency bands, and time windows of interest

**Fig. 2:**
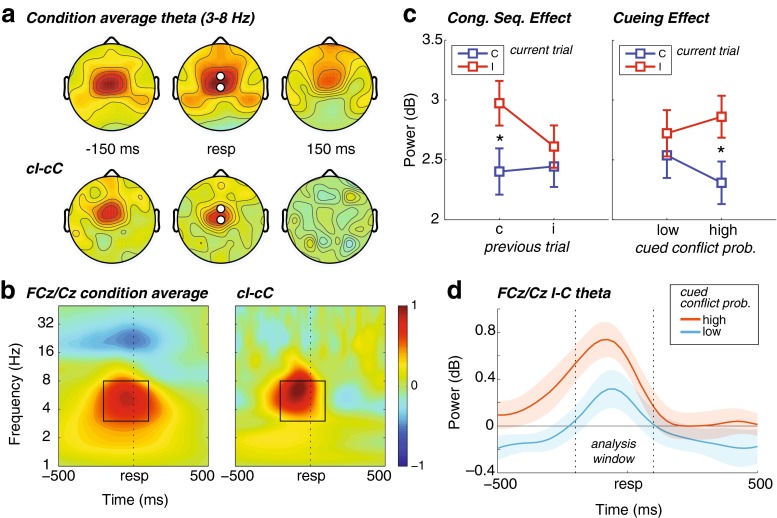
Response-locked oscillatory power results. (**a**) Condition average (top) and conflict-related (bottom) topographical maps of theta (3–8 Hz) power, revealing a midfrontal (FCz/Cz) hotspot of activity. cI, incongruent trial preceded by congruent trial; cC, congruent trial preceded by congruent trial. Black-and-white disks denote the electrodes plotted in panel **b**. (**b**) Time–frequency maps at the electrodes highlighted in panel **a** (pooled), showing condition average (left) as well as conflict-related (right) activity in theta power around the response. The black-outlined squares show the time–frequency window of interest used for the ANOVA. (**c**) Average midfrontal theta activity in the time window of interest for congruent (C) and incongruent (I) trials as a function of previous trial congruency (left) and the cued conflict probability (right). Error bars represent ±1 *SEM*. (**d**) Conflict-related (I–C) midfrontal theta power plotted over time for high (top curve) and low (bottom curve) cued conflict probability. The vertical dashed lines denote the ANOVA time window selected in panel **b**. Shaded area = ±1 *SEM*

To a priori select channels and time–frequency windows of interest for further statistical analyses, we took the following approach: First, we computed condition-average (i.e., averaging over groups, congruencies, and conflict probabilities) topographical maps of response-locked theta (3–8 Hz) power, for several time points around the response. This revealed a clear locus of theta activity around electrodes Cz and FCz; these electrodes were pooled as one midfrontal electrode pair. Next, we computed a condition-average time–frequency map of this midfrontal region, which revealed a clear “hotspot” of theta-band (3- to 8-Hz) activity from –200 to 100 ms relative to the response, which we selected as our main time–frequency window of interest (see Fig. 2a and b). Note that because of condition averaging, this selection procedure was orthogonal to, and thus not biased by, potential condition differences between (1) congruent or incongruent trials, (2) the conflict probability conditions, and (3) interactions between these factors.

**Fig. 3:**
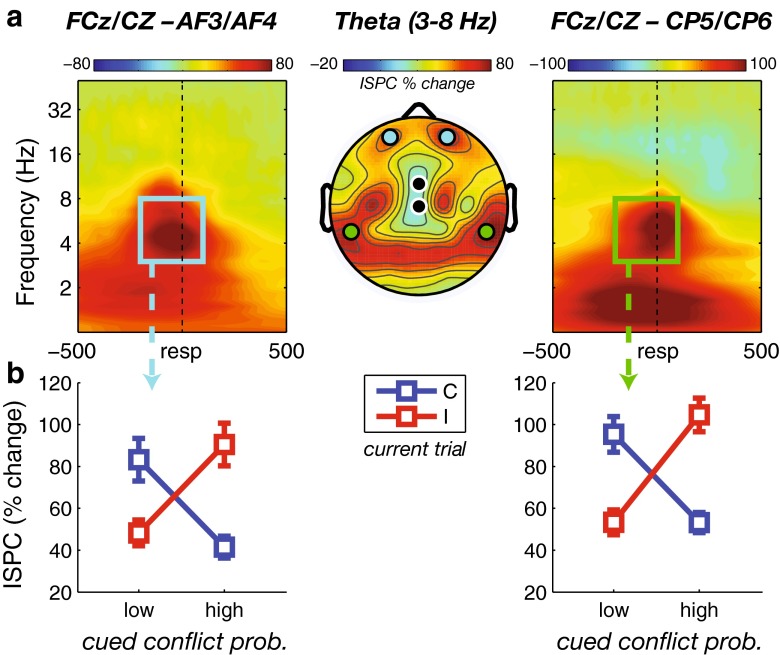
Response-locked intersite phase clustering (ISPC) results. (**a**) Topographical map of condition-averaged midfrontal-seeded theta ISPC (middle), revealing connectivity between midfrontal and lateral prefrontal (AF3/AF4, left) sites and posterior (CP5/CP6, right) sites around the response. The outline box on the left and the top electrode disks in the map denote the time–frequency–space selection for lateral frontal connectivity; the box on the right and the bottom electrode disks denote time–frequency–space selection for posterior connectivity. The black disks in map depict the seed region of midfrontal FCz/Cz sites. (**b**) Average theta ISPC in the time windows denoted in panel **a** (as indicated by the arrows), plotted for congruent (**c**) and incongruent (I) trials as a function of cued conflict probability, for the respective target regions (left, AF3/AF4; right, CP5/CP6) showing connectivity with the midfrontal seed region. Error bars represent ±1 *SEM*

For ISPC analyses, the midfrontal electrodes FCz/Cz now served as “seeds” of targeted synchronization in the theta band. First, we plotted a condition-averaged topographical map of ISPC over the same time–frequency window as used for the power analysis. This revealed two bilateral regions of interest of functional connectivity with our midfrontal electrode pair: one over lateral frontal sites (AF3/AF4), and one over lateral parieto-occipital sites (CP5/CP6). Subsequent time–frequency maps of ISPC between these two target regions and the midfrontal region indeed showed strong connectivity in the theta frequency band around the time of the response (Fig. 3a). Importantly, the selection procedure of target electrodes for this seeded-synchrony analysis was data driven, and unbiased because of our condition-averaging approach (see above). Although interregional connectivity could be expected, given the communicative cognitive control signals between, for example, MFC and DLPFC (see the introduction), the exact electrodes were determined through this approach.

**Fig. 4:**
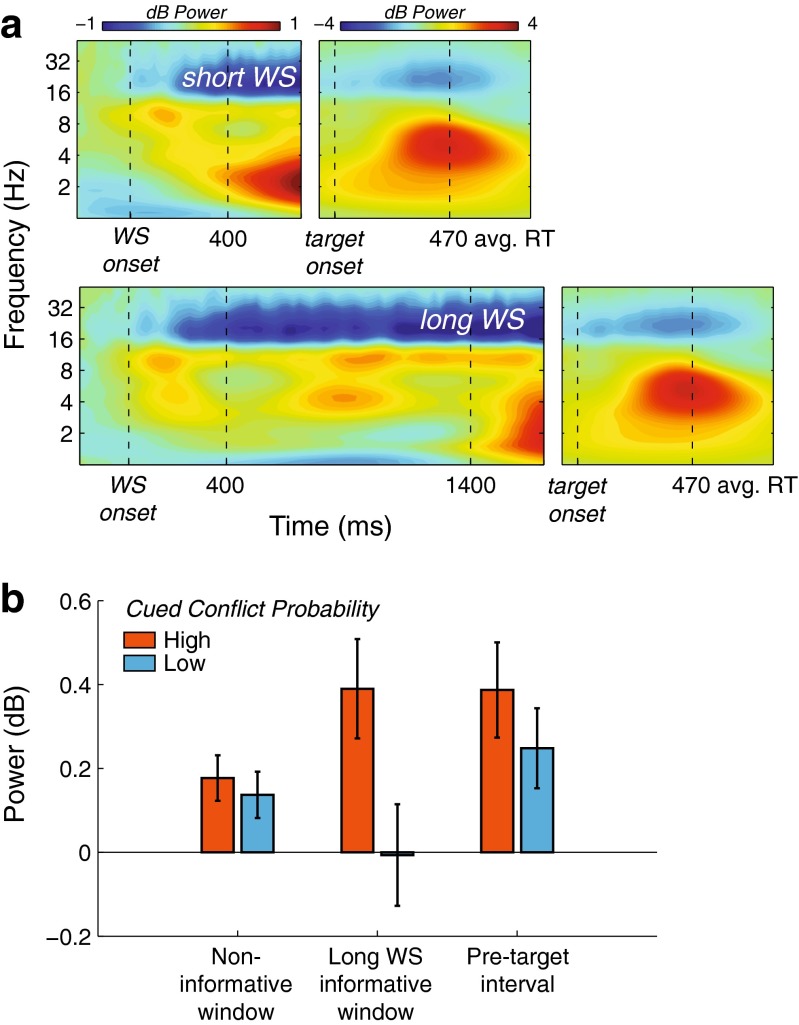
Cue-locked pretarget power results. (**a**) Condition-average time–frequency maps of midfrontal power, plotted separately for short-warning-signal (WS; top) and long-WS (bottom) trials. Activity is plotted separately for the pretarget (left; time relative to WS onset) and posttarget (right; time relative to target onset) periods due to scaling differences. Vertical dashed lines demarcate the time windows of interest, reflecting short- and long-WS onset and offset, target onset, and average RT. (**b**) Bar plot of average midfrontal theta power during three time windows of interest, separately for high and low cued conflict probability in the noninformative window (0–400 ms post-WS), informative window (400–1,400 ms post-WS; long-WS trials only), and pretarget interval (–300 to 0 ms pretarget). Error bars represent ±1 *SEM*

In addition to reactive (i.e., response-locked) control mechanisms, we were also interested in cue-related, proactive (i.e., during the WS/PTI) control processes. To this end, we used the same frequency band (3–8 Hz) and electrode pair (FCz/Cz) as for the response-locked power analysis, and looked for pretarget dynamics of theta power during the WS and the PTI. We reasoned that during the first 400 ms of either the short or the long WS, subjects could not infer its predictive value. This time window could thus always be regarded as noninformative. The PTI, on the other hand, could always be regarded as an informative time window: After WS offset (irrespective of whether this was after 400 or 1,400 ms), subjects could infer whether conflict probability was high or low. The interval from 400 to 1,400 ms after WS onset provided only for the long-WS trials an informative cue about conflict probability, as well: For the late-conflict group, a long WS cued high conflict probability, whereas for the early-conflict group, a long WS cued low conflict probability. Importantly, evaluating the effect of conflict probability during this informative time window of the long-WS trials was by definition a between-group comparison. First, we computed the average midfrontal theta-band activity over these three time windows (0–400, 400–1,400, and 1,400–1,700 ms relative to WS onset), separately for high and low conflict probability. Second, as an exploratory analysis, we additionally plotted separate midfrontal time–frequency maps of the short- and long-WS trials, averaged over the two groups and thereby over cued conflict probability, to identify different time–frequency windows of interest (Fig. 4a). On the basis of these plots, we computed the average activity in the alpha (8–14 Hz) and beta (15–25 Hz) frequency bands during the same noninformative, informative, and PTI time windows, and performed the same statistical analysis (see below) as for theta activity.

**Fig. 5:**
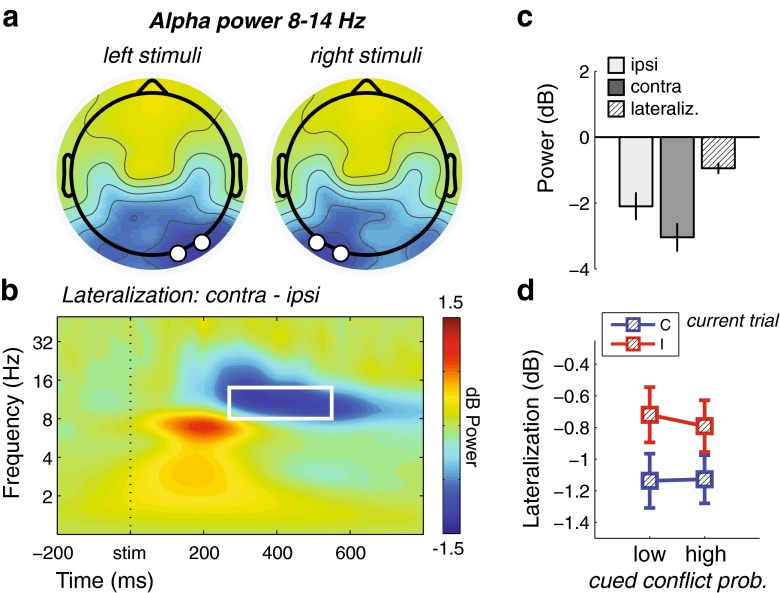
Stimulus-locked alpha lateralization results. (**a**) Topographical maps of alpha (8–14 Hz) power 300–600 ms poststimulus, plotted separately for stimuli presented to the left and right of fixation, showing contralateral posterior alpha suppression. The black-and-white disks denote the electrode selection for further analysis: PO8/O2 for left stimuli, and PO7/O1 for right stimuli. (**b**) Time–frequency map of contralateral minus ipsilateral activity (averaged over left and right stimuli), confirming modulation in alpha-band activity as a function of stimulus location. The white-line box denotes time–frequency window used for the ANOVA. (**c**) Bar plots of ipsilateral (light gray), contralateral (dark gray), and contralateral–ipsilateral (lined) posterior alpha power. (**d**) Average power of the time–frequency window denoted in panel **b**, plotted for congruent (C) and incongruent (I) trials as a function of cued conflict probability. Error bars represent ±1 *SEM*

Finally, we tested whether cued conflict probability could have an effect on spatial attention toward the stimulus location, since this was the conflicting dimension in the Simon task. Spatial attention has been shown to elicit posterior alpha (8–14 Hz) suppression contralateral to the attended hemifield (Sauseng et al., 2005; Thut, Nietzel, Brandt, & Pascual-Leone, 2006). Thus, we analyzed epochs on the basis of presentation side (left/right), conflict probability (high/low), and target congruency (congruent/incongruent). Topographical maps of alpha-band power at several posttarget time windows revealed a clear decrease in alpha activity at electrodes PO7/O1 for right-presented stimuli, and PO8/O2 for left-presented stimuli (Fig. 5a). Time–frequency maps of contralateral minus ipsilateral activity of these channels (i.e., the average of PO7/O1 – PO8/O2 for right stimuli, and PO8/O2 – PO7/O1 for left stimuli, collapsed across conflict probability and congruency) confirmed this alpha suppression effect to be present during a 270- to 550-ms window (Fig. 5b). Lateral alpha power was thus defined as the average power at these posterior channels and time–frequency window.

### Statistical analyses

The aim of our analyses were (1) to test whether the temporal cue could be used to reduce the conflict effect, and (2) to assess whether conflict-related theta-band activity, reflecting control processes (Cavanagh & Frank, 2014; Cohen, 2014), would already have commenced during the conflict-predicting intervals, thus not exclusively being present during the time of the response. We therefore analyzed both behavior (as reaction times [RTs] and accuracy) and brain activity (power and ISPC) using repeated measures analysis of variance (ANOVA) with the within-subjects factors of interest Current Trial Congruency (congruent vs. incongruent) and Conflict Probability (high vs. low). Since the target congruency of a previous trial has been shown to influence behavior on a current trial (e.g., Egner, 2007; Gratton et al., 1992; Kerns et al., 2004), we also included Previous Trial Congruency (congruent vs. incongruent) as a factor, to account for confounding influences of previous congruency. Furthermore, although we were not interested in a possible effect of WS duration, per se, we included the between-subjects factor Group as well, so that possible group differences or interactions would indicate stronger temporal-cueing effects for longer or shorter WS durations. In all ANOVAs, when the assumption of sphericity did not hold, the Greenhouse–Geisser correction was applied, although we report the original degrees of freedom for ease of interpretation. Post-hoc dependent-samples *t* tests were performed to explore any interaction effects. Error and posterror trials were excluded from all analyses, except for the behavioral analysis of accuracy. For brain activity, we computed power and ISPC for each subject averaged over the time–frequency windows and electrodes, as specified above.

In the pretarget analysis, we conducted a separate independent-samples *t* test for activity during the informative window of the long WS, in which the late- and early-conflict groups were directly compared, reflecting a comparison of high and low conflict probability, respectively. In the posterior alpha lateralization analysis, we used a repeated measures ANOVA with the within-subjects factors Laterality (contra vs. ipsi), Conflict Probability (high vs. low), and Current Trial Congruency (congruent vs. incongruent), and the between-subjects factor Group.

To assess the relation between our behavioral and electrophysiological effects, we performed Spearman rank-correlation tests. For the correlations, a single measure was computed for the difference in the conflict effects (incongruent [I] minus congruent [C]) induced by conflict probability (*conflict-cueing effect*): (I – C)_high_ – (I – C)_low_. This measure was computed for RTs, power, and ISPC (for the RT analysis, within-subjects standardized RTs were used in order to make the measure more comparable between subjects; for power and ISPC, the decibel and percent change corrected values were used, respectively). On the basis of the previous literature (Cohen & Donner, 2013; Cohen & Ridderinkhof, 2013), we expected to find positive correlations, and thus set the statistical test of significant correlation to be one-tailed.

Finally, to evaluate the time course of one of our significant ANOVA effects of EEG power (see below), we performed time-wise permutation testing with cluster-based thresholding, as a correction for multiple comparisons. Specifically, the permutation test transformed the average condition difference power value at each time point from decibels into a *z* value with respect to a null distribution of surrogate condition difference values, obtained by swapping condition labels for a random half of subjects at each of 1,000 permutations. The resulting *z* scores were thresholded at *p* < .05. With an additional 1,000-iteration permutation test, a distribution of cluster sizes of contiguous significant time points under the null hypothesis of no condition difference was computed, and only clusters that exceeded the 95th percentile of this distribution were retained.

### Single-trial regression analysis

The above-described analyses were all performed on the basis of trial-averaged data. Additionally, it would be revealing to take into account within-subjects intertrial variability (Cohen & Cavanagh, 2011; Pernet, Sajda, & Rousselet, 2011): In this way, one could more readily infer that proactive control triggered by conflict-predicting time intervals, and reactive control triggered by actual conflict, are dynamic, single-trial adaptive processes. To this end, we assessed whether the online neural dynamics directly reflected (i) anticipated conflict during the conflict-predicting intervals, (ii) experienced conflict at time of the target, and (iii) the validity of the conflict expectation at the time of the target.

First, we computed, for each subject, single-trial midfrontal theta power, averaged over three time windows: the PTI (–300 ms to target onset) as the conflict-predicting interval, the response-related interval (–200 to 100 ms, response-locked), and the noninformative time window (0–400 ms post-WS-onset) as a control interval. Second, we determined the trial-type labels: Each trial had a WS that predicted either low or high conflict probability, a target that was either congruent or incongruent, and the predictive cue could either match (e.g., high conflict probability followed by an incongruent trial) or not match (e.g., high conflict probability followed by a congruent trial) the eventual level of conflict. Next, we tested whether single-trial pretarget and response-related theta power was a reliable predictor of these trial-type labels. For each subject, we fitted three logistic regression models for the average single-trial power values of the three different time windows. In turn, each resulting *β* term consisted (in addition to the intercept) of three regression weights that corresponded to the degree to which theta power predicted each trial-type label. For each subject, these regression weights were binarized as 1 if the regression weight indicated that increased theta power over trials predicted a high-conflict-probability cue, an incongruent trial, or a match between cue and congruency, and as 0 if increased theta power predicted a low-conflict-probability cue, a congruent trial, or a nonmatch between cue and congruency. The regression weights were tested at the group level against .5 (reflecting chance-level predictive value of the binarized regression weights) using one-sample Mann–Whitney *U* (also known as the *Wilcoxon rank sum*) nonparametric *t* tests, which were considered significant if they exceeded a Bonferroni-corrected threshold of *p* < .0167 (i.e., .05 divided by the three time windows tested). See Cohen and Donner (2013) for a more detailed description of this approach. The rationale behind the binary recoding of regression weights was that because condition labels are binary, a continuous beta weight of theta power predicting such a binary variable would be less intuitive. We confirmed that repeating the analyses with continuous regression weights led to the same pattern of results.

### Follow-up behavioral experiments

To assess whether the effects of the temporal cues extended to nontemporal cues, we conducted three additional behavioral (no EEG) experiments. The tasks and procedures were identical to those of the EEG experiment, apart from the type of cue used. All of the task parameters and statistical analyses of behavioral performance were in line with those from the EEG experiment, except where noted.

#### Follow-up Experiment 1: nontemporal implicit cueing

In this experiment, the cue consisted of a horizontal black bar (height 0.25 dva) presented at fixation. The cue duration was always 900 ms (i.e., the average of the 400- and 1,400-ms WS durations in the EEG experiment), and was followed by the PTI and target. Crucially, the width of the horizontal bar cue was either 0.5 or 1.26 dva. In the short-conflict condition, a short (vs. long) horizontal bar predicted the upcoming trial to be incongruent (vs. congruent) with 80 % validity. In the long-conflict condition, the cues had the opposite meanings. Randomly, ten subjects were assigned to the short-conflict condition, and the other ten were assigned to the long-conflict condition. The rationale behind this experiment was that the width (spatial length) of the bar should be analogous to the duration (temporal length) of the WS from the EEG experiment, in serving as a cue. However, here the prediction of the cue could be inferred upon its presentation (because the difference in width could be instantaneously perceived), whereas in the EEG experiment this could only be inferred after a certain time had passed (because the WS was perceptually identical in all trials, except for its duration).

#### Follow-up Experiment 2: probabilistic semantic cueing

In this experiment, the task was identical to the task of the first follow-up experiment, except that we used the semantically meaningful words “HARD” and “EASY” as the conflict-predicting cues (with the same 80 % validity), instead of the horizontal bars. These cues allowed for a complete within-subjects design (i.e., no Group factor was needed to counterbalance the cue-probability mapping across subjects). This experiment started with two practice blocks: a noncue version of the Simon task (with an intertrial interval of 1,000 ms) of 25 trials, and a 100 %-validity practice block of 25 trials using the word cues. The experiment consisted of six blocks of 100 trials each, separated by self-paced breaks.

#### Follow-up Experiment 3: informative versus uninformative cueing

In this experiment, the task was identical to the task of the second follow-up experiment, except for the cue validity and the inclusion of a neutral cue. Here, the word “HARD” was *always* followed by an incongruent trial, the word “EASY” was *always* followed by a congruent trial, and the word “NEUTRAL” was followed 50 % of the time apiece by an incongruent and by a congruent trial. Because the informative cues were 100 % valid in this experiment, this design allowed for a comparison of the conflict effects following the informative cues versus following a neutral, uninformative cue. Thus, in this experiment the factor Cue Type had the two levels informative versus uninformative, instead of high versus low conflict probability as in the other experiments.

## Results

### Behavioral results from EEG experiment

On average, the subjects responded correctly on 92.76 % (*SD* = 3.04) of the trials, with an average response speed of 467.40 ms (*SD* = 57.12). On correct trials (also excluding posterror trials), RTs increased significantly on incongruent as compared to congruent trials [*F*(1, 28) = 32.56, *p* < .001], reflecting the classic conflict effect induced by the irrelevant spatial dimension of the stimulus. In addition, we found a significant interaction between current and previous trial congruency [*F*(1, 28) = 26.92, *p* < .001]. As can be seen in Fig. 1c (upper panel), the conflict effect on RTs decreased when the preceding trial was incongruent [*t*(29) = 1.32, *p* = *.*12], relative to when it was congruent [*t*(29) = 7.18, *p* < .001]. This replicates earlier findings of the congruency sequence effect (Egner, Ely, & Grinband, 2010; Gratton et al., 1992). Both the conflict effect and the conflict sequence effect were present in accuracy, as well: Subjects performed worse on incongruent than on congruent trials [*F*(1, 28) = 8.74, *p* = *.*006], and this decrease in performance was attenuated when the previous trial was incongruent [*F*(1, 28) = 11.88, *p* = *.*002].

Importantly, our manipulation of pretarget conflict cueing based on temporal information showed an unexpected finding (Fig. 1c, lower panel). When the WS duration predicted high conflict probability, the conflict effect on RTs increased, as compared to when the WS duration predicted low conflict probability [*F*(1, 28) = 32.56, *p* < .001]. For accuracy, we found a similar, though nonsignificant, effect [*F*(1, 28) = 3.78, *p* = *.*062]. Moreover, these effects did not depend on whether the subjects performed the early- or the late-conflict condition, as is indicated by the absence of an interaction with the between-subjects factor Group [RT: *F*(1, 28) = 3.23, *p* = *.*083; accuracy: *F* < 1]. In other words, when conflict could be anticipated on the basis of the duration of a fixation cross (irrespective of whether this predicting interval was short or long), this hampered conflict resolution.

We further explored this negative effect of cueing on conflict by examining RT distributions through delta plots, in which the conflict effect (the difference in RTs between incongruent and congruent trials) is plotted as a function of average RT. This approach has been shown to be sensitive to variations and dynamics in conflict effects that are otherwise lost in regular trial-average scores (Ridderinkhof, 2002; van den Wildenberg et al., 2010). Figure 1d shows that throughout the RT distribution, the conflict effect was stronger for high- than for low-conflict-probability cueing [Congruency × Conflict Probability interaction, *F*(1, 29) = 24.0, *p* < .001]. Irrespective of conflict probability, on the other hand, the conflict effect became reduced with longer RTs [Congruency × RT Bin interaction: *F*(3, 27) = 75.95, *p* < .001], as is evidenced by a negative slope of the delta plot [*F*(1, 29) = 142.4, *p* < .001], which is consistent with previous reports of the Simon task and is interpreted as the selective suppression of location-based response capture (Burle, van den Wildenberg, & Ridderinkhof, 2005; Ridderinkhof, 2002). Since congruency, RT bin, and conflict probability did not interact [*F*(3, 27) = 1.99, *p* = *.*16], selective suppression was not influenced by the temporal cueing of conflict.

The effect of conflict probability on current trial congruency did not further interact with previous trial congruency, which suggests that the mechanism that was driving the conflict-cueing effect was different from the mechanism that mediated conflict adaptation (Alpay et al., 2009; Egner, 2007). This is an important finding, because conflict adaptation, as reflected by the CSE, can be considered a form of anticipatory control, as well (see the introduction). Furthermore, we observed no main effect of conflict probability, and in general the two groups did not differ in RTs (all *F*s < 1). The early-conflict group did perform better than the late-conflict group in terms of accuracy [*F*(1, 28) = 4.62, *p* = *.*04]. An additional analysis of the possible influence of the WS duration of the preceding trial on the RT at the current trial showed that there was no main effect of current and previous trial WS duration (all *F*s < 1), nor an interaction between these factors [*F*(1, 28) = 1.96, *p* = *.*17], signifying that our manipulation of different WS durations, which resulted in different intervals between successive targets, did not affect general, nonspecific preparation to respond to these targets (Los & Agter, 2005).

Together, these behavioral dynamics point to an effect of conflict cueing that is opposite from what has been reported previously (Wendt & Kiesel, 2011): When the probability of conflict could be inferred on the basis of temporal information, behavioral responses to conflict further deteriorated.

### Response-related EEG time–frequency power

On the basis of a condition-orthogonal contrast of response-related oscillatory power against baseline, we chose a two-channel (FCz and Cz) midfrontal pair of electrodes for our main analyses (see the Materials and method section). As is shown in Fig. 2a and b, this electrode pair showed an increase in activity in the theta band (3–8 Hz) over a time window of –200 to 100 ms surrounding the buttonpress. Average activity in this time–frequency window concurred with our behavioral findings described above (Fig. 2c). First, we found a conflict-related (i.e., I – C) increase in midfrontal theta power [*F*(1, 28) = 26.39, *p* < .001], which was stronger when the previous trial was congruent than when it was incongruent [*F*(1, 28) = 20.75, *p* < .001], corroborating previous findings (e.g., Cohen & Donner, 2013; Pastötter, Dreisbach, & Bäuml, 2013). Second, the midfrontal conflict-related theta power was modulated by conflict probability [*F*(1, 28) = 10.82, *p* = *.*003], independent of previous trial congruency (*F* < 1): Conflict-related theta power was stronger after high than after low conflict probability, mimicking the (unexpected) behavioral cueing effect.

**Fig. 6:**
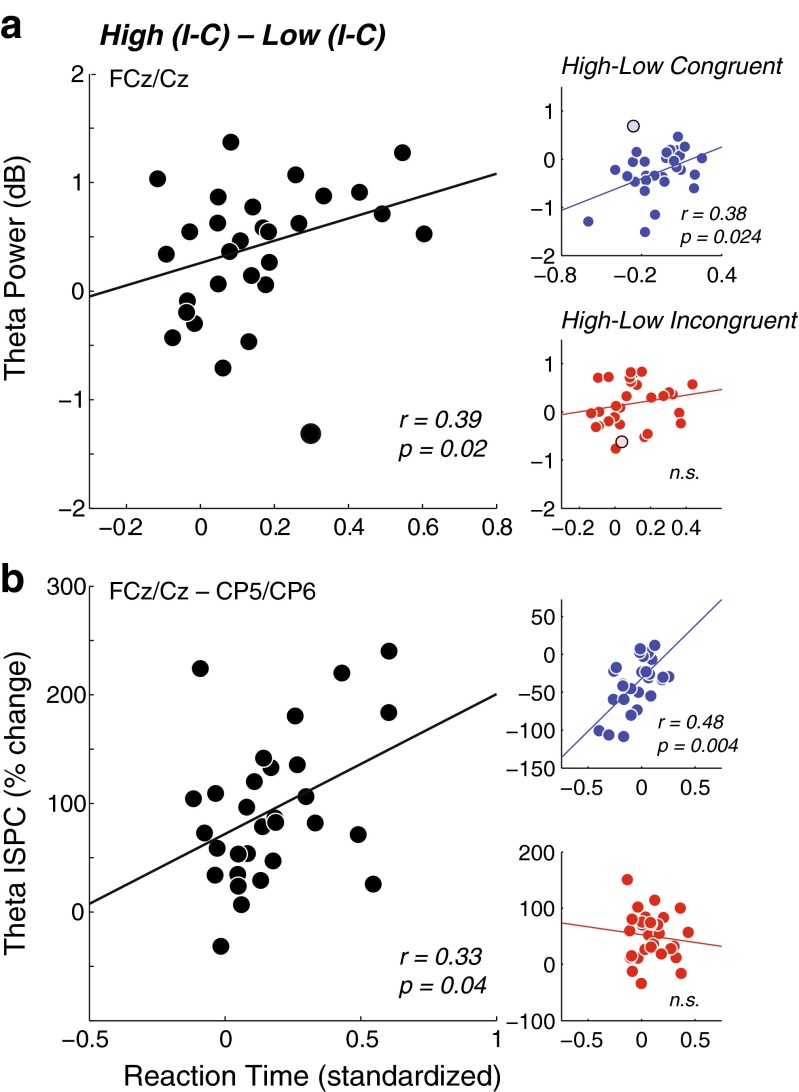
Cross-subject correlations. (**a**) Subject-specific average midfrontal (FCz/Cz) theta power (from the ANOVA for the time–frequency window; see Fig. 2a and b), as a function of the subject-specific RT. Left: Correlation of conflict-related difference scores (I–C), modulated by cued conflict probability (high–low). The black-bordered gray disk depicts one outlier subject. Right: Correlations of congruent (top) and incongruent (bottom), modulated by cued conflict probability (high–low). The same outlier subject is highlighted, as in the left plot. (**b**) Same as in panel **a**, for theta ISPC between midfrontal and posterior (CP5/CP6) sites

Indeed, the cueing effect on theta power correlated across subjects with the cueing effect on behavior (Fig. 6a): Subjects who exhibited more conflict-related behavioral slowing after a high- than after a low-conflict-probability cue also showed a stronger conflict-related increase in midfrontal theta power after a high- than after a low-conflict-probability cue (*r* = .31, *p* = *.*049 [*r* = .39, *p* = *.*020 when excluding one marked outlier]). Interestingly, this cross-subject correlation seemed to be driven mostly by the effect of conflict probability on congruent trials (*r* = .33, *p* = *.*037 [*r* = .38, *p* = *.*024, when excluding the earlier outlier]), and less on incongruent trials (*r* = .26, *p* = *.*084 [*r* = .25, *p* = *.*097, when excluding the outlier]).

No further interaction effects with group occurred, nor any main effects of conflict probability or (previous trial) WS duration (all *p*s > .1). Thus, in line with the behavioral findings, temporal cueing of conflict showed a specific effect of increased local conflict-related theta-power dynamics, again independent from the actual duration of the cue.

Because we restricted this analysis to an a-priori-chosen time window, we next explored the time course of this effect, which is illustrated in the line plot in Fig. 2d. The effect of current trial congruency on midfrontal theta clearly dropped to zero before and after the response. However, over a longer time window of –500 to 50 ms around the response, conflict-related theta power was significantly elevated when high conflict probability rather than low conflict probability was cued (as revealed by time-wise permutation testing with cluster-size thresholding). Thus, the modulation of conflict-related midfrontal theta power by conflict cueing was already present around target onset, and may even have extended to a pretarget time window (average RTs were below 500 ms). A pretarget conflict-related effect may seem odd, given that actual conflict is not yet known, but careful inspection of Fig. 2d shows that around this time, the two lines that are separated on the basis of cued conflict probability together average out to zero. Thus, this preresponse—and possibly pretarget—effect is most likely driven by the cued likelihood of upcoming conflict; this interpretation was confirmed below (see the Cue-related pretarget EEG time–frequency power section).

### Response-related intersite phase clustering

In addition, we were interested in whether conflict cueing modulated interregional connectivity, as well. To test this, we computed intersite phase clustering (ISPC; see the Materials and method section) between the midfrontal electrode pair used for the power analysis (here, thus, used as “seed”) and all other electrodes, which revealed theta-band synchronization between this region and a bilateral prefrontal region (AF3/AF4), as well as a bilateral centro-parietal region (CP5/CP6; see Fig. 3a). Importantly, the selection of these electrodes was data driven and unbiased, because this selection was orthogonal to potential condition differences. Although midfrontal connectivity with lateral prefrontal electrodes could be expected on the basis of earlier findings (e.g., Cohen & Cavanagh, 2011), the finding of midfrontal–centro-parietal connectivity was not hypothesized a priori. Using the same time–frequency windows that were used for the power analysis, a similar repeated measures ANOVA revealed a marginal effect of current trial congruency for the lateral prefrontal region only [*F*(1, 28) = 4.11, *p* = *.*052; centro-parietal: *F*(1, 28) = 1.36, *p* = *.*25], where incongruent trials elicited stronger ISPC than congruent trials. Previous and current congruency did not further interact [lateral prefrontal: *F*(1, 28) = 2.04, *p* = *.*17; centro-parietal approaching significance: *F*(1, 28) = 3.79, *p* = *.*062].

However, we observed a strong interaction between conflict probability and current trial congruency [lateral prefrontal: *F*(1, 28) = 66.17, *p* < .001; centro-parietal: *F*(1, 28) = 95.19, *p* < .001; Fig. 3b], which, as in the power and behavioral results, did not interact with group (*F* < 1). Moreover, whereas a cue predicting high conflict probability was followed by stronger ISPC for incongruent than for congruent trials [lateral prefrontal: *t*(29) = 7.00, *p* < .001; centro-parietal: *t*(29) = 6.89, *p* < .001], a cue predicting low conflict probability was followed by a reverse effect, of stronger ISPC after congruent than after incongruent trials [lateral prefrontal: *t*(29) = 4.77, *p* < .001; centro-parietal: *t*(29) = 5.93, *p* < .001]. This could not be attributed to differences in trial number, because ISPC values tend to be higher for low trial counts, whereas we found the reverse pattern.

Similar to the power results, the cueing effect on centro-parietal (FCz/Cz–CP5/CP6) theta-band connectivity correlated with the cueing effect on behavior (Fig. 6b): Subjects that showed stronger conflict-related RT slowing after a high- than after a low-conflict-probability cue also showed a stronger conflict-related increase in theta ISPC after a high- than after a low-conflict-probability cue (*r* = .33, *p* = *.*036). Again, as with power, this correlation was stronger in degree when considering only congruent trials (*r* = .48, *p* < .004), and was absent for incongruent trials (*r* = –.04, *p* = *.*59). The theta ISPC between midfrontal and lateral prefrontal regions (AF3/AF4) showed no significant correlations (all *p*s > .1).

### Cue-related pretarget EEG time–frequency power

The results above provide evidence that the conflict-predicting temporal cue affected both behavioral performance and the associated brain dynamics, though in the opposite direction from the one expected. To examine whether this could be explained by changes in pretarget cue-related activity, we plotted time–frequency power locked to the WS onset, for both short- and long-WS trials, collapsed over groups (i.e., averaged over conflict probability). As can be seen in Fig. 4a, this revealed modulations in the theta, alpha (8–14 Hz), and beta (15–25 Hz) bands. We hypothesized that this would give us a neural measure of conflict anticipation, because of a possible modulation of activity between cue and target, as a function of conflict probability.

To test this, we first restricted our analysis to the theta band, and computed midfrontal theta power in three time windows of interest: the first 400 ms over both short- and long-WS trials (the “noninformative” time window), 400–1,400 ms for long-WS trials only (the “informative” time window), and the PTI for both short- and long-WS trials (see the Materials and method section for the rationale behind these time window labels). As expected, during the noninformative window, midfrontal theta power did not differ between the high- and low-conflict probability conditions [*F*(1, 28) = 0.96, *p* = *.*33], nor did it interact with group [*F*(1, 28) = 0.12, *p* = *.*74]. However, during the informative window, subjects for whom the long WS predicted high conflict probability showed stronger midfrontal theta activity than did subjects for whom the long WS predicted low conflict probability [*t*(29) = 2.38, *p* = *.*024]. During the subsequent PTI, this difference between high and low conflict probability persisted, for both short and long WSs [i.e., a main effect of conflict probability: *F*(1, 28) = 4.91, *p* = *.*035, without an interaction with group, *F* < 0.1]. Thus, pretarget midfrontal oscillatory dynamics showed a preparatory effect of conflict cueing with a hazard function characteristic: When the conditional probability of conflict increased (vs. decreased) over time, given that the WS had not yet ended, midfrontal theta power concomitantly increased (vs. decreased).

Second, we explored alpha- and beta-band power, because the condition-averaged time–frequency maps of long and short WS also showed activity in these bands (see Fig. 4a). During the same time windows used for the theta-band analysis, alpha power was not modulated by any of our factors, nor by their interactions (all *p*s > .1). However, beta power showed an interaction between conflict probability and group during the PTI [*F*(1, 28) = 4.39, *p* = *.*048], in which a post-hoc independent-samples *t* test revealed that after a low-conflict-probability cue, beta suppression was stronger for the early-conflict group than for the late-conflict group [*t*(28) = 2.54, *p* = *.*017]. That is, beta suppression was stronger after a long WS than after a short WS, because the long WS was a low-conflict-probability cue for the early-conflict group, and the short WS was a low-conflict-probability cue for the late-conflict group. This result can be explained by an effect of duration on beta suppression, which typically develops in strength over time when preparing for a motor response (de Jong, Gladwin, & ’t Hart, 2006). Interestingly, this effect was absent when both a short and a long WS indicated a high probability of conflict [*t*(28) = 1.51, *p* = *.*14].

In sum, these results provide evidence for a neural signature of conflict anticipation (increase in pretarget midfrontal theta power), triggered by temporal cues that predict conflict.

### Within-subjects single-trial regression

**Fig. 7:**
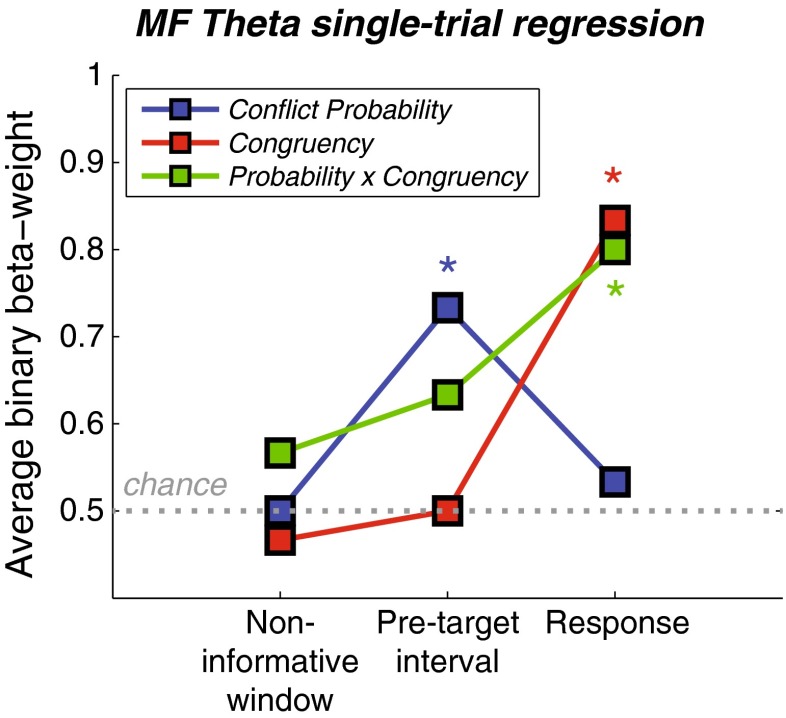
Single-trial midfrontal theta regression results. Plotted are subject-average binary beta weights (0 = negative prediction, 1 = positive prediction) as a function of time window, shown separately for the trial-type predictors conflict probability, congruency, and their interaction. Regression models including these three predictors were fitted per time window, on the single-trial midfrontal theta power averaged over the respective time window. The gray dotted horizontal line denotes chance prediction by the model. Colored asterisks denote significant regressions between single-trial theta and trial type (Wilcoxon rank sum *t* tests, *p* < .017)

Conflict anticipation triggered by conflict-predicting time intervals, and reactive control triggered by actual conflict, should manifest at the single-trial level. We hypothesized that the degree of midfrontal theta power would fluctuate over the course of the trial, depending on whether (i) the temporal cue predicted conflict, (ii) the trial subsequently contained a conflict target, and (iii) the prediction of the cue was valid. To this end, we performed a logistic regression analysis (see the Materials and method section), in which we used single-trial midfrontal theta power averaged over three time windows (the noninformative window, the PTI [see Fig. 4b], and the response-related window used for the general power analysis [see Fig. 2b]) to predict these condition labels. The results are shown in Fig. 7, where on the *y*-axis the percentage of subjects is shown who exhibited a positive relationship between theta and the condition label. A Wilcoxon signed-rank test corroborated the trial-averaged group-level results. First, theta activity during the noninformative window remained at the chance prediction level (all *p*s > .4). Second, during the PTI, theta power predicted the type of cue (*p* = *.*011), where stronger theta was associated with high-conflict-probability cues. Finally, during the response-related window, theta power predicted the actual congruency (*p* < .001), where stronger theta power was associated with incongruent trials. Interestingly, stronger response-related theta was also predictive of the cue–congruency match (*p* = *.*001), reflecting at the single-trial level the group-level interaction between conflict probability and current trial congruency described above. For example, when a high-conflict-probability cue was followed by an incongruent trial, such a trial was likely to result in stronger theta activity; and similarly, a low-conflict-probability cue followed by a congruent trial was also likely to result in stronger theta.

In sum, this analysis revealed evidence of single-trial conflict anticipation based on temporal information, expressed in midfrontal theta-band activity. Moreover, the single-trial activity corroborated the seemingly contradictory cueing effect around the response, of increased conflict-related midfrontal theta power when this conflict could be anticipated.

### Target-related lateralized alpha

We next tested whether the cueing of conflict could have affected low-level processing of stimulus features as well. In the Simon task, the feature that is of particular interest is the target location, as this is the irrelevant dimension that leads to response conflict. We reasoned that posterior alpha suppression contralateral to side of stimulus presentation would be a strong correlate of lateralized attentional processing (Sauseng et al., 2005; Thut et al., 2006). The spatial location of the stimulus indeed elicited a strong contralateral alpha suppression over parieto-occipital regions [*F*(1, 28) = 39.70, *p* < .001], around 300 ms after target onset. This means that, for example, when a stimulus was presented on the left side of the screen, alpha power suppression was relatively stronger over right parieto-occipital sites than over left parieto-occipital sites (see Fig. 5a–c) This alpha lateralization effect was modulated by current trial congruency [*F*(1, 28) = 15.43, *p* = *.*001], in which congruent trials elicited stronger alpha lateralization than incongruent trials [*t*(29) = 3.67, *p* = *.*001; Fig. 5d]. However, conflict probability did not interact with stimulus location (*F* < 1), nor was there a three-way interaction between conflict probability, current trial congruency, and stimulus location (*F* < 1). Importantly, these null findings suggest that cueing conflict did not affect bottom-up processing of sensory features, as it did not modulate a neural index of spatial attention (Sauseng et al., 2005; Thut et al., 2006). On the other hand, these results do show how spatial attention was reduced (as indicated by relatively less lateralized alpha power suppression) after targets of which the spatial location was incongruent with the required response. Given the absence of an effect of cueing, however, this spatial attention effect could be regarded reactive rather than proactive.

### Behavioral results of follow-up experiments

We set out to further pin down this surprising finding by means of three follow-up experiments, in which we varied the symbolic and probabilistic nature of the cues (see Materials and method above) in the same Simon task.

#### Follow-Up Experiment 1: nontemporal cueing

In short, this task was identical to the temporal-cueing task, except that the cues consisted of horizontal bars of equal duration, with the width forming a cue for conflict probability. As expected, this task resulted in slower responses on incongruent than on congruent trials [*F*(1, 18) = 22.79, *p* < .001], and this conflict effect was reduced when the previous trial was incongruent as compared to when it was congruent [*F*(1, 18) = 42.98, *p* < .001]. The same pattern was found for accuracy (both *p*s < .05).

**Fig. 8:**
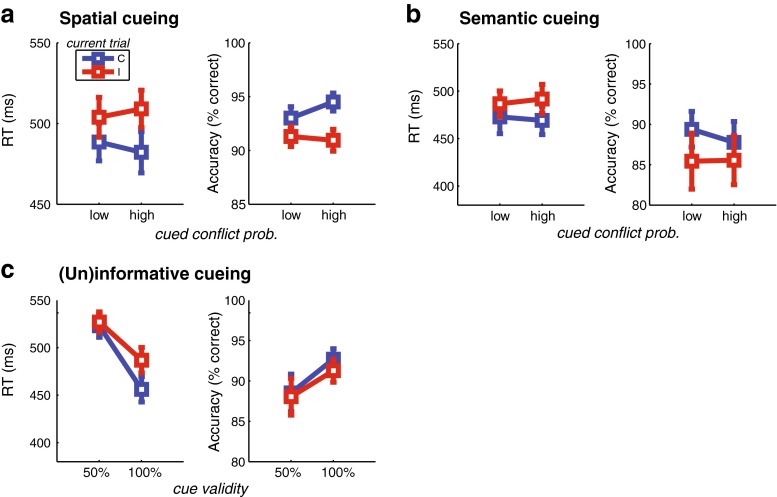
Behavioral results of the follow-up experiments. In all plots, RTs and accuracy are shown for congruent (C) and incongruent (I) targets at the current trial as a function the cue. Error bars represent ±1 *SEM*. (**a**) Spatial cueing with horizontal bars predicting low (20 %) or high (80 %) conflict probability, depending on spatial length. (**b**) Semantic cueing, in which the words “EASY” and “HARD” predicted low (20 %) or high (80 %) conflict probability. (**c**) Here the semantic cues were 100 % valid, as compared with a neutral cue (the word “NEUTRAL”) that was 50 % valid

Importantly, this task elicited the same contradictory effect of conflict cueing [*F*(1, 18) = 4.64, *p* = *.*045; Fig. 8a]. Similar to the manipulation of time intervals as cues, when the nontemporal symbolic information (i.e., the width of a bar) cued high conflict probability, the conflict effect in RTs increased, as compared to when the cued conflict probability was low [*t*(19) = 2.09, *p* = *.*050]. Again, this effect did not interact with group [*F*(1, 18) = 2.22, *p* = *.*15], nor with previous trial congruency (*F* < 1). For accuracy, we did not observe an interaction between the current trial congruency and conflict probability in this experiment [*F*(1, 18) = 1.11, *p* = *.*31].

#### Follow-Up Experiment 2: probabilistic semantic cueing

To investigate whether the symbolic nature of the cue is an important variable in the results above, we repeated the same experiment, except that the cues comprised words that were semantically informative about conflict (e.g., “HARD”), while still predicting conflict with 80 % certainty. Although we found the same general conflict and sequence effects (all *p*s < .05), any effect of cueing was absent. That is, no main effect of conflict probability emerged [RT: *F*(1, 14) = 1.36, *p* = *.*26; accuracy: *F* < 1], nor any interaction with current and previous trial congruency (all *F*s < 1; Fig. 8b).

#### Follow-Up Experiment 3: informative versus uninformative cueing

Introducing an uninformative cue (the word “NEUTRAL”) that predicted conflict with 50 % probability, together with the “HARD” and “EASY” words, which now predicted the upcoming congruency as informative cues, with 100 % validity, showed a pattern of results that replicated earlier findings (Strack et al., 2013). First, subjects responded faster [*F*(1, 14) = 22.88, *p* < .001] and more accurately [*F*(1, 14) = 9.60, *p* = *.*008] when the cues were informative than when they were uninformative (Fig. 8c). Second, the conflict effect for RTs was higher following informative than following uninformative cues [*t*(14) = 3.58, *p* = *.*003], which was reflected by an interaction between the factors Cue Type and Current Trial Congruency [*F*(1, 14) = 12.80, *p* = *.*003]. In other words, people benefited from the informativeness, or validity, of the cue in preparing for incongruent trials [*t*(14) = 3.91, p = .002], but this effect was even stronger when preparing for congruent trials [*t*(14) = 5.12, *p* < .001]. These interactions were not present for accuracy (all *F*s < 1).

## Discussion

In this study, we hypothesized that if temporal information derived from between-trial intervals correlated with the probability of future instances of conflict (Wendt & Kiesel, 2011), subjects could use this contingency to prepare for upcoming conflict through anticipatory proactive control (Correa et al., 2009). We predicted that the conflict effect (increased RTs and decreased accuracy for incongruent vs. congruent trials) would be reduced when conflict could be expected on the basis of the duration of a “warning signal” (an intertrial fixation cross). Surprisingly, the present data point to the exact opposite conclusion. In fact, the conflict effect was present only when conflict probability was cued to be high (80 % incongruent trials), and disappeared when conflict probability was cued to be low (80 % congruent trials).

Although this result was contrary to our predictions and in sharp contrast with previous findings of conflict-reducing effects of temporal cueing (Wendt & Kiesel, 2011), this pattern of behavioral results was internally consistent with several distinct manifestations of EEG dynamics, and generalized to other task settings. First, we obtained strong evidence of increased conflict-related midfrontal theta-band (3–8 Hz) power, and stronger conflict-related interregional theta synchrony, specific to situations of high conflict likelihood. These neurophysiological underpinnings of cognitive control (Cohen, 2014) correlated across subjects with the behavioral cueing effect.

Second, in addition to temporal cues, *nontemporal*, *symbolic* cues (here, horizontal bars of different width) increased the conflict effects as well. Only when the cues were semantically meaningful words that were 100 % valid (e.g., the word “hard” always appeared before an incongruent trial), we found a behavioral benefit with respect to a neutral, uninformative (50 % valid) cue. However, this effect was most pronounced for congruent trials.

### Frontal theta dynamics reflect both anticipatory proactive and posttarget reactive control

Our general EEG results of increased frontal theta power as well as interregional phase synchrony after conflict are in accordance with a growing body of findings that have tied frontal theta-band activity to various cognitive control processes, including conflict adaptation (Cohen & Cavanagh, 2011; Pastötter et al., 2013), error processing (Luu, Tucker, & Makeig, 2004; van Driel et al., 2012), task switching (Cunillera et al., 2012), and reinforcement learning (Cavanagh et al., 2010; van de Vijver, Ridderinkhof, & Cohen, 2011). An important contribution of this article to the literature is that midfrontal theta increases can already be observed before a conflict target, elicited by a conflict-predicting cue. Moreover, by using time intervals as such cues, we observed that these anticipatory dynamics waxed and waned around temporal windows during which the cue became informative. These intertrial events were perceptually identical except for duration, showing that midfrontal theta activity can, in addition to reflecting posttarget control processes, be linked to an endogenously generated conflict anticipation process that is based on an internal representation of time. The surprising finding is that this conflict anticipation did not produce adaptive behavior; it is thus questionable whether these anticipatory processes could be regarded as proactive *control*. However, a recent study has shown that under certain circumstances, cue-induced cognitive control can indeed impair rather than facilitate behavior (Bocanegra & Hommel, 2014).

Although we observed the “classic” effect of response-locked conflict-related increases in midfrontal theta power after target onset (Cohen, Ridderinkhof, Haupt, Elger, & Fell, 2008; Nigbur, Ivanova, & Stürmer, 2011), this effect emerged only when conflict probability was cued to be high. This cue–conflict interaction was present at the single-trial level and paralleled the behavioral findings, and both effects correlated across subjects. Moreover, the theta effect was present well before the response, and thus may be a result of the pretarget cue-related increase in midfrontal theta. Although this interpretation is post hoc, it may provide an explanation of our unexpected findings: It is possible that the cue-related theta effect is, in terms of the underlying mechanism, qualitatively different from conflict-related theta (Cohen, 2014). This view is in accordance with several studies demonstrating that anticipatory activity in the medial frontal cortex can be independent from, and can dampen, subsequent conflict-related medial frontal activity (Aarts, Roelofs, & van Turennout, 2008; Brown, 2009; Ide, Shenoy, Yu, & Li, 2013; Luks, Simpson, Dale, & Hough, 2007; Oliveira, Hickey, & McDonald, 2014). In our task, these processes may have interfered around the time of the response (i.e., during action selection), resulting in less efficiently applied reactive control. Varying the interval between cue and target, thereby teasing apart these processes in time, may be a way to further investigate this hypothesis.

In addition to conflict-related local power, we reported interregional theta phase synchrony between midfrontal and lateral frontal (Cohen & Cavanagh, 2011; Hanslmayr et al., 2008), as well as posterior (Anguera et al., 2013; Cohen & van Gaal, 2013), sites. This large-scale functional connectivity was stronger after incongruent than after congruent targets, exclusively following a cue that was associated with high conflict probability; after a low-conflict-probability cue, interregional theta-band connectivity reversed, becoming stronger after congruent than after incongruent targets. This seems inconsistent with a recently proposed interpretation of frontal theta phase synchrony reflecting the top-down implementation of control in response to general signals of “surprise” (Cavanagh & Frank, 2014). That is, the more obvious hypothesis, that unexpected (i.e., surprising) events should require relatively more control, would predict the exact opposite. Nonetheless, our connectivity effects were remarkably strong and were consistent with the local theta power effects, in terms of both the group-level effects and the cross-subject correlations.

From an anatomical perspective, mid–lateral frontal theta synchrony has been proposed to reflect MFC–DLPFC functional connectivity, which increases after conflict has been encountered (Cohen & Ridderinkhof, 2013). The current axiom in the cognitive control literature is that the MFC *monitors* for possible instances of conflict, and upon conflict detection, communicates the need for increased control to the DLPFC, which further *implements* control through top-down signals to motor and task-relevant sensory areas (Botvinick et al., 2004; Botvinick, Nystrom, Fissell, Carter, & Cohen, 1999; Kerns et al., 2004; MacDonald et al., 2000; Ridderinkhof et al., 2010; Ridderinkhof, Ullsperger, et al., 2004; Ridderinkhof, van den Wildenberg, Segalowitz, & Carter, 2004). However, direct regulatory top-down signals from MFC to guide behavior in situations of conflict have also been observed (Cohen et al., 2009; Danielmeier et al., 2011; Kennerley, Walton, Behrens, Buckley, & Rushworth, 2006; Ridderinkhof, Ullsperger, et al., 2004), suggesting a more integrative function of the MFC (Shenhav, Botvinick, & Cohen, 2013). Our findings of theta synchrony between midfrontal and posterior parietal regions are in accordance with this view. This debate notwithstanding, implemented control signals, be they directly from MFC activity, or in concert with DLPFC, should increase after uncertain, high-conflict situations; here, we found that these signals became stronger when a prediction (incongruent or congruent) was met. We now turn to possible alternative explanations for this unexpected finding.

### Temporal cues and attention to time

One possible, albeit speculative, explanation of our findings could be that time intervals are special in serving as cues triggering specific anticipatory activity (Sperduti, Tallon-Baudry, Hugueville, & Pouthas, 2011; but see the next paragraph). Given that the intertrial fixation stimuli were valid predictors only with respect to their *duration*, subjects may have increased their attention to the passage of time *especially* when this duration signaled conflict. Research on “temporal orienting” has shown that attention to time can boost bottom-up processing of perceptual information (Cravo, Rohenkohl, Wyart, & Nobre, 2011, 2013; Jepma, Wagenmakers, & Nieuwenhuis, 2012; Nobre et al., 2007; Rohenkohl, Cravo, Wyart, & Nobre, 2012), which can interfere with cognitively controlled action selection (Correa, Cappucci, Nobre, & Lupiáñez, 2010), presumably because the irrelevant conflicting information is processed to a stronger degree. Thus, in our study, the temporal cues that predicted high conflict likelihood may have resulted in more instead of less conflict, by enhancing sensory processing of the irrelevant (conflicting) spatial location of the stimulus, through increased attention to time. However, this explanation would have predicted that neural activity related to spatial processing would increase after cues that signaled high conflict probability. In contrast, we found that contralateral posterior alpha suppression decreased after incongruent targets; these alpha dynamics were not affected by cueing. This finding is consistent with lateralized alpha power reflecting an index of top-down control over spatial attention (Klimesch, 2012; Sauseng et al., 2005; Thut et al., 2006), and should here be interpreted as *reactive*, because the top-down signal was observed *after* conflict was encountered.

Moreover, our behavioral follow-up experiment in which we used nontemporal, symbolic instead of temporal cues also argues against the explanation of time intervals being special. That is, when the pretarget stimuli comprised horizontal bars that differed in spatial length instead of duration (i.e., “temporal length”), subjects were again not able to use these cues to improve behavior, as was evidenced by a similar increase in conflict effects after high-conflict-probability cueing. Nonetheless, the use of time as a source of information for abstract inferences (such as predicting future conflict likelihood) has received little emphasis (Appelbaum et al., 2012; Wendt & Kiesel, 2011), and thus the underlying processes of encoding this temporal information as a usable contextual cue remain largely unknown.

### Alternative explanations

Our behavioral results initially seem to be in contrast with those from studies that have shown beneficial effects of cueing in conflict tasks (Crump et al., 2006; Fischer, Gottschalk, & Dreisbach, 2014; Ghinescu, Schachtman, Stadler, Fabiani, & Gratton, 2010; Gratton et al., 1992; King, Korb, & Egner, 2012). However, a careful examination of this literature provides some leverage for relating our study to previous findings.

First, several studies have reported the effect of cueing to be most prominent on congruent trials (Aarts et al., 2008; Alpay et al., 2009; Klein & Ivanoff, 2011; Stoffels, 1996; Strack et al., 2013; Wühr & Kunde, 2008). This is consistent with our findings. For example, we found that the behavioral effect of cueing correlated with both midfrontal theta power and midfrontal–parieto-occipital theta phase synchrony, only for congruent and not for incongruent trials. Second, some studies have failed to show clear benefits of cueing in general (Goldfarb & Henik, 2013; Luks et al., 2007), or on incongruent trials specifically (Strack et al., 2013), and, depending on the specific task settings, have even reported opposite effects (Alpay et al., 2009; Wühr & Kunde, 2008). Third, the types of cues and conflict tasks have differed widely across studies, while often lacking a detailed rationale as to why these settings were chosen. For example, although some have argued that a sufficiently long time interval is required between cue and target in order to generate an expectation about upcoming conflict (Correa et al., 2009; Monsell, 2003), others have shown that varying the cue–target interval does not modulate the cueing effect (Wühr & Kunde, 2008). Indeed, the observation that features of the target itself can trigger conflict adaptation “on the fly” (King et al., 2012; Lehle & Hübner, 2008) argues for a fairly rapid and flexible form of proactive control.

Over and above the timing of the cue and target, the nature of the conflict paradigm itself may be crucial in determining whether cues can be used for proactive control. Superficially, conflict tasks such as the Stroop, flanker, and Simon tasks appear very similar, since they all produce qualitatively similar behavioral conflict effects; however, the exact neurocognitive processes that produce these effects may be markedly different (Hommel, 2011). In the Simon task, the task-irrelevant dimension of the target location needs to be processed before the task-relevant dimension of color can be processed. That is, the target needs to be located before the color can be determined. In contrast, in the flanker task, the task-relevant information, which is the central target, can be processed directly, without processing the task-irrelevant peripheral distractors. It might be that conflict anticipation only translates to improved proactive control in conflict paradigms in which the location of the conflicting, task-irrelevant dimension is known a priori. Indeed, pretarget cueing of conflict has been shown to result in behavioral improvement in the flanker task (Correa et al., 2009).

In the Stroop task, the task-irrelevant and task-relevant stimulus dimensions share the same location (e.g., the word RED shown in green). A previous study (Appelbaum et al., 2012), however, showed that indeed, when these dimensions are untangled in time (e.g., the word RED first appears in white, and only subsequently changes color) and when these moments in time were predictable, performance improved. Thus, the temporal and spatial predictability of the conflicting stimulus dimension seems important. Applying this argument to our findings, it could be that expecting conflict on the basis of a conflict-predicting cue results in further *increased* instead of decreased conflict, because one is not able to a priori suppress spatial attention when the conflicting stimulus location is unknown. In other words, conflict is increased by both the cue and the target, in an additive manner. However, it is harder to envisage how this would explain *reduced* conflict after a *low*-conflict-probability cue.

Another alternative explanation for increased behavioral conflict effects after cues signaling high conflict probability is that these cues may trigger a generalized cautious response mode of proactive slowing, possibly through an increased decision threshold. However, several features of the data argue against this account. First, *general* proactive slowing after a warning cue that signals high conflict likelihood would still predict a decreased conflict effect. That is, responses to low-conflict (congruent) trials are usually fast, which would predict that responses to these trials would be most affected by a cautious response mode by becoming slower. In contrast to this prediction, we found that responses to congruent trials after cues that predicted high conflict probability became *faster*. Second, high-conflict trials by themselves are already characterized by slower responses; thus, a proactive cautious response mode should affect these trials less; in contrast, we found that responses to incongruent trials became *even slower* when these were cued, as compared to when congruent trials were cued. Third, one could argue that, by means of a speed–accuracy trade-off (see Egner, 2007), a cautious, conservative response mode would be expressed both by slower RTs *and* higher accuracy. In other words, the effects on RTs should be similar to the effects on accuracy. In contrast to this prediction, we observed an inverse pattern in accuracy as compared to RTs, in relation to cueing.

### From symbolic, probabilistic cueing to semantic, deterministic cueing

Our follow-up experiments suggest three additional variables in conflict cueing that may prove important in disambiguating our findings. First, the *semantic level* of the cue may influence whether and how proactive control can develop (Fischer et al., 2014; Umbach, Schwager, Frensch, & Gaschler, 2012). In our study, time intervals and horizontal bars had, in contrast to the words “HARD” and “EASY,” no a priori relationship with conflict. Using the word cues, we found no cueing effect when these cues were probabilistic (i.e., predicting with 80 % validity upcoming conflict), which is in accordance with results from another study (Alpay et al., 2009). Other studies have varied in the semantic level of the cue. For example, it can be argued that a red cross and a green checkmark (Correa et al., 2009) contain intrinsic information about conflict to a stronger degree than do contextual target features (e.g., the color of a flanker stimulus; Vietze & Wendt, 2009). In addition, the semantic level of arbitrary symbols can change depending on the task instructions and training (Ghinescu et al., 2010), which may alter task strategies and conscious experience of (pretarget) conflict. It has been shown that the latter influence can modulate the behavioral conflict effect in an opposite direction (Desender, Van Opstal, & Van den Bussche, 2014).

Second and third, the *validity* of the cue may be important (Lai & Mangels, 2007; Vossel, Thiel, & Fink, 2006), which may or may not require the inclusion of a *neutral* cue. In our study, changing the cueing paradigm from probabilistic (80 % validity) to deterministic (100 % validity) resulted in behavioral improvements, especially on congruent trials and in comparison with neutral cues, replicating previous findings (Alpay et al., 2009; Strack et al., 2013). This effect can be more easily explained: Neutral cues predict uncertainty, resulting in more cautious response strategies for both congruent and incongruent trials. On the other hand, valid, explicit cues result in faster responses in general, which favors the faster “direct” route through which the irrelevant stimulus dimension (location) is processed, over the slower “deliberate” route that processes the relevant stimulus dimension (color) (Ridderinkhof, 2002; van den Wildenberg et al., 2010). Thus, although conflict-predicting cues speed up behavior, this increased impulsivity results in a stronger conflict effect. Interestingly, a study by Cavanagh, Zambrano Vazquez, and Allen (2012) did not show effects of cue validity on behavior, and they observed a very modest *decrease* of midfrontal theta EEG dynamics for informative relative to uninformative cues. In designing future studies involving probabilistic temporal and spatial cues, researchers may wish to include a neutral cue, because this could provide more insight into whether the obtained costs of cueing may be related to either impulsivity or cautiousness.

### Conclusions

We found a behavioral cost of time-based conflict anticipation, which was mirrored in frontal theta EEG dynamics and replicated in other cue and task settings. Previous findings on pretarget cueing have been mixed, and some of our results can be linked to these anomalies. However, how exactly informative cues can hurt instead of help performance in terms of what is cued needs further research.
